# Editorial: Rising stars in parasite and host: 2023

**DOI:** 10.3389/fcimb.2024.1509107

**Published:** 2024-12-02

**Authors:** Glenn A. McConkey

**Affiliations:** Faculty of Biological Sciences, University of Leeds, Leeds, United Kingdom

**Keywords:** parasite, protozoan, host-pathogen, editorial, molecular

We are at a time in science with great prospects for significant contributions from rising stars in parasitology. This field is being enhanced by major discoveries and developments in molecular and cell biology; particularly the rapid advances in genomics, other “omics” and high-throughput technologies. The application of molecular techniques from PCR to single-cell sequencing has been instrumental in advances in the detection, tracking and characterization of protozoan parasites. The knowledge gained has been utilized to investigate the evolution, epidemiology and biology of these microscopic organisms.

In recent years, the field of molecular biology and genetics of parasites has witnessed remarkable evolution, particularly in the realms of genomics and high-throughput next-generation sequencing (NGS). The advent of NGS technologies has revolutionized our understanding of the complexity and functionality of their genomes. These advances have enabled the comprehensive analysis of many parasite genomes, leading to the identification of numerous genetic variations and their unique characteristics.

The integration of NGS with ‘omics’ approaches, such as transcriptomics, proteomics, and metabolomics, has facilitated a more holistic view of parasite biological systems. This synergy has been pivotal in unraveling the intricate networks of gene expression regulation, protein interactions, and metabolic pathways. The application of NGS in omics studies has also accelerated the discovery of markers for parasite diagnosis, epidemiology, and therapeutic target identification.

Molecular parasitology has greatly benefited from these technological strides, allowing for the exploration of genetic landscapes in diverse parasites with unprecedented resolution. The use of NGS in molecular genetics has provided insights into the mechanisms of parasite biology, life cycle stages and host interactions. The field of genomics has expanded our knowledge of numerous genomes with the Welcome Sanger Institute listing 25 annotated parasitic protozoan genomes. Efforts to further expand the number of known protist genomes have led to the sequencing of 629 species (Genomes Online Database (GOLD), Joint Genome Institute) and the Protist 10,000 Genomes Project (https://ngdc.cncb.ac.cn/p10k/). This project is currently sequencing 1101 new species and has the goal of sequencing 10,000 of the 60,000-200,000 protist species. This has had significant implications for understanding parasite evolution, metabolism, and host evasion.

Advances in molecular biology, genetics, and genomics have also been instrumental in the response to global health challenges. Indeed, the COVID-19 pandemic showed how these technologies could be applied on a global scale with technologies such as RT-PCR and NGS, which played a crucial role in the rapid sequencing of the SARS-CoV-2 genome. These techniques, applied in hundreds of testing facilities, were essential to the development of diagnostic tests, vaccines and treatments. Their applications have permitted strain detection, monitoring of viral evolution, and tracking.

In this Research Topic a series of articles written by rising stars in parasitology apply the transformative developments in molecular biology, molecular genetics and genomics driven by advances in NGS and omics technologies, to expand our understanding of parasitic infections and parasite biology. These breakthroughs have not only deepened our understanding of parasites at the molecular level, but have also opened new avenues of scientific research, with far-reaching implications for disease diagnosis and treatment.

The articles in this Research Topic study parasitic protozoa in the phyla Euglenozoa, Metamonada and Apicomplexa with the early-branching kinetoplastid *Trypanosoma brucei* and *Giardia* and Hemosporidia, respectively. Apicomplexa is a large phylum that includes *Plasmodium*, the agent responsible for malaria. *Plasmodium* species are host-specific with five species (out of hundreds described) infecting humans. The coccidial Apicomplexa *Cryptosporidium, Cystoisospora* and *Toxoplasma* (which escape the gut to become systemic) are zoonotic and can be transmitted between animals and humans. These coccidia are intestinal parasites as are *Giardia* and *Blastocystis* which are studied in the articles. *Trypanosoma brucei*, the agent responsible for African sleeping sickness, represents the flagellated kinetoplastids.

Among the Research Topic in *Rising Stars in Parasite and Host: 2023* articles apply molecular biology techniques for the identification, epidemiology, evolution, and diagnosis of parasitic infections. Three of the articles (Yun et al.; Musa et al.; Mei et al.) use molecular techniques involving DNA amplification and sequencing for the detection and characterization of parasitic protozoa with applications in diagnosis, screening, and epidemiology. Mutant libraries of *T. brucei* were developed for unbiased scanning of residue contributions in one study (McDermott et al.).

Articles in the Research Topic employ molecular parasitology to investigate differences in life cycle stages and host interactions. Post-transcriptional mRNA editing investigations in *T. brucei* found life cycle stage differences between stages in the vertebrate host and stages in the insect vector in one study (McDermott et al.). *T. gondii* infection prompts a systemic immune response but parasites that reach immunoprivileged tissues convert to slow-growing tissue cyst forms that evade removal, establishing a chronic infection. The early immune responses of a type III lineage strain of *T. gondii* were described in another study. Immune markers using qRT-PCR found that rather than the association of strain type with particular markers, the immune response was associated with the mortality rate of the isolate (Uzelac et al.). In highly complex and poorly understood host-parasite interactions, *T. gondii* induces host manipulation and behavioral changes in animals that are advantageous in the transmission of the parasite to the definitive host (termed ‘fatal feline attraction). Changes at the molecular level in microflora, neurotransmission and immune activation were evaluated in the final paper of this series (Prandovszky et al.).

The intestinal protozoan *Blastocystis*, a member of the Stramenopiles, responsible for diarrhea and gastrointestinal problems has traditionally been diagnosed microscopically by fecal smear. In this series, Mei et al. developed isothermal polymerase amplification with lateral flow detection as a rapid, sensitive, specific detection by dipstick. The test was consistent with the sensitivity of previous PCR assays of fecal samples and was highly specific for this pathogen. The assay is rapid, easy and does not require microscopy expertise.

Detection and differentiation of the intestinal parasites *Cryptosporidium, Cystoisospora*, and *Giardia duodenalis* and species identification were completed by PCR and sequencing of fecal samples in one of the studies (Yun et al.). Molecular phylogenetic analysis identified subspecies of *Giardia* in assemblages A, B and C. By this approach zoonotic protozoan parasites were identified and characterized in feline populations.

Molecular detection and identification were again applied in a third paper in the series. This time avian infections to differentiate species of Hemosporidia. The initial development of genus-specific detection of avian *Plasmodium* infection by PCR with species identification by sequencing was conducted in 1996 (McConkey et al., 1996) This has grown as a method for species identification. In the study in the series presented here, a nested PCR approach was developed using broad Hemosporidia primers for the initial PCR with subsequent nested PCR for genus-specific amplification (Musa et al.) PCR detection is challenging in birds due to the nucleated red blood cells that increase the background. Lineages of *Plasmodium*, *Hemoproteus* and *Leucocytozoa* were detected in a large collection of blood samples from passerine birds by the nested PCR protocol and sequencing.

To uncover the functional characteristics of a protein central to RNA editing in the parasitic protozoa trypanosomes, a high throughput mutational screening of mutant libraries was developed. As an early divergent group, characterization in comparative, bioinformatic analysis is hampered by low homology to known proteins; necessitating experimental mutant analysis. RNA editing in kinetoplastid mitochondria involves uridine insertions and deletions to generate open reading frames in mRNAs. In this study, thousands of variants were tested for their effect on parasite growth in bloodstream forms (McDermott et al.). This permitted the detection of amino acid residues that are functionally involved but not apparent from conservation in sequence alignments for this highly divergent organism. RNA editing variants differed in their phenotype between vertebrate bloodstream and insect host stages of their life cycle.

Host interactions were further investigated in the study of early immune responses in type III *T. gondii* (Uzelac et al.). The genus *Toxoplasma* contains a single species but this species is composed of several strains with the three archetypes, type I, type II, and type III, which are distributed globally. The types differ in virulence with type I being virulent in mice and types II and III being non-virulent. Type II strains dominate human infections and are most commonly associated with AIDS patients. Most prevalent in immunocompetent individuals with severe ocular toxoplasmosis are type I genotype parasites. Type I is also associated with severe congenital toxoplasmosis. Strain-specific differences in immune responses were recently reviewed by Saeij’s group (Mukhopadhyay et al., 2020). Type I and III strains induce a much milder pro-inflammatory response than type II strains. Type III strains, which do not express the parasitic rhoptry protein ROP18, are sensitive in mice to cellular immune-related genes that destroy parasitic vacuoles and hence rely on the virulence factor ROP16 to dampen the initial immune response early after infection. In the study in this series, isolates of different virulences were examined for cytokine levels and an immune marker by qRT-PCR found elevated systemic IFNγ in a high mortality strain while it was down-regulated in isolates with low mortality rates in mice.

Arguably one of the most interesting facets of biology, at the interface of the parasite with the host, is the altered host behavior induced by infection. This effect of *T. gondii* on mammals was presented by Prandovszky et al. During stages of chronic infection, when the parasite is encysted in neurons in the host brain, changes in the behavior of the intermediate host have been observed (reviewed in Chapter here) (Cairney and A., 2024). Notably, infected rodents exhibit increased exploratory activity, delayed arousal and loss of their innate fear of cat odors (Webster, 1994; Vyas et al., 2007; Ihara et al., 2016; Alsaady et al., 2019). Behavioral changes have been observed in a variety of infected intermediate hosts ranging from rodents, wolves and chimpanzees to humans (Flegr et al., 2002; Poirotte et al., 2016; Meyer et al., 2022). Seroprevalence of *T. gondii* has been associated with schizophrenia in numerous studies (Sutterland et al., 2015). Alterations in neurotransmission have been observed with changes in neurotransmission. The levels of catecholamines and dopaminergic and noradrenergic signaling are altered during chronic infection (Xiao et al., 2014; Alsaady et al., 2019; Boillat et al., 2020; Cromar et al., 2022; Tedford et al., 2023). Changes have also been seen in the distribution of the γ-aminobutyric acid biosynthetic enzyme GAD67, decreased glutamate transporter in astrocytes, and dendritic spine loss.

The number and types of contributors to these neurological and behavioral changes during *T. gondii* infection remain unclear. Immune activation, parasite products, neuronal signaling, and changes in the microflora have been posited. Intriguingly, even mice infected with an attenuated strain of *T. gondii* maintain their changes in behavior when the parasite is no longer detectable in the brain; implicating a long-term or permanent host change (Ingram et al., 2013), which could support the involvement of immune dysfunction, epigenetic changes in the host brain, or microflora dysbiosis induced by infection. Indeed, elevated levels of pro-inflammatory cytokines correlated with increased behavioral changes (Boillat et al., 2020). Recently, *T. gondii* infection was found to induce DNA methylation changes in the vasopressin receptor gene and the key gene for norepinephrine synthesis in the brain and, importantly, paracrine signaling of the epigenetic changes via extracellular vesicles to uninfected bystander neurons was observed (Hari Dass and Vyas, 2014; Tedford et al., 2023). Evidence of gut microflora changes has been observed during chronic infection; with enrichment of Bacteroidetes (Prandovszky et al., 2018). Acute infection of the intestine induces dysbiosis and an imprint is maintained during chronic infection with multiple studies reporting an enrichment in the *Verrucomicrobia*. In the review in this Research Topic, several possible contributors are discussed with a focus on the potential role/s of gut microbial dysbiosis.
